# Catalytic Hydrodechlorination of 4-Chlorophenol by Palladium-Based Catalyst Supported on Alumina and Graphene Materials

**DOI:** 10.3390/nano13091564

**Published:** 2023-05-06

**Authors:** Jintae Jeon, Yuri Park, Yuhoon Hwang

**Affiliations:** Department of Environmental Engineering, Seoul National University of Science and Technology, Seoul 01811, Republic of Korea

**Keywords:** catalytic hydrodechlorination, palladium, alumina, graphene, graphene oxide sand composite (GOSC), 4-chlorophenol

## Abstract

Hydrodechlorination (HDC) is a reaction that involves the use of hydrogen to cleave the C−Cl bond in chlorinated organic compounds such as chlorophenols and chlorobenzenes, thus reducing their toxicity. In this study, a palladium (Pd) catalyst, which is widely used for HDC due to its advantageous physical and chemical properties, was immobilized on alumina (Pd/Al) and graphene-based materials (graphene oxide and reduced graphene oxide; Pd/GO and Pd/rGO, respectively) to induce the HDC of 4-chlorophenol (4-CP). The effects of the catalyst dosage, initial 4-CP concentration, and pH on 4-CP removal were evaluated. We observed that 4-CP was removed very rapidly when the HDC reaction was induced by Pd/GO and Pd/rGO. The granulation of Pd/rGO using sand was also investigated as a way to facilitate the separation of the catalyst from the treated aqueous solution after use, which is to improve practicality and effectiveness of the use of Pd catalysts with graphene-based support materials in an HDC system. The granulated catalyst (Pd/rGOSC) was employed in a column to induce HDC in a continuous flow reaction, leading to the successful removal of most 4-CP after 48 h. The reaction mechanisms were also determined based on the oxidation state of Pd, which was observed using X-ray photoelectron spectroscopy. Based on the results as a whole, the proposed granulated catalyst has the potential to greatly enhance the practical applicability of HDC for water purification.

## 1. Introduction

Most chlorinated compounds are harmful as they have adverse effects on the environment and human health due to the presence of chlorine in their structure [1]. Chlorophenols (CPs) are one of the organic compounds that are produced widely within the manufacturing industry (e.g., for pharmaceuticals, plastics, and dyes) and as disinfection by-products from the residual chlorine after chlorination; thus they are present in a wide range of natural environments [2,3,4]. It is known that CPs are environmentally harmful owing to its toxicity, persistent nature, and strong bioaccumulation with high water solublity and stability [5]. CPs are also known as precursors of persistent organic pollutants (POPs) such as hexachlorobenzene (HCB), polychlorinated biphenyls (PCBs), polychlorinated dibenzo-*p*-dioxins (PCDDs), polychlorinated dibenzofurans (PCDFs), and pentachlorobenzene (PeCB), which are listed under the Stockholm Convention because of their carcinogenic, mutagenic, and cytotoxic properties [6,7]. Therefore, CPs need to be removed from industrial wastewater. 

Traditionally, post-treatment technologies such as incineration, adsorption, and oxidation have been employed for CP removal. However, the incineration process can generate by-products such as PCDDs and PCDFs that are more toxic than chlorophenol itself [8,9]. CP removal was effectively achieved by adsorption with activated carbon, which is the most commonly used adsorbent. Adsorption capacity is dependent on the solution pH, contact time, reaction temperature, the surface area of the adsorbent, and the functional groups present on the adsorbent surface [10]. Although adsorption is one of the most efficient methods for removing water contaminants, highly toxic by-products can be generated during the regeneration of used adsorbents, limiting the range of potential conventional and non-destructive processes for the treatment of CPs [11,12]. Advanced oxidation processes (AOPs) are a more effective option for removing CPs, but they require high energy input and generate more toxic chlorinated species and recalcitrant products [13,14,15,16,17] if complete mineralization is not achieved. 

Catalytic hydrodechlorination (HDC), which was first examined under the mild reaction conditions by Hoke et al. (1992), has been suggested as a promising and non-destructive method that offers in-situ dechlorination without toxic by-products [18,19,20,21]. For liquid-phase HDC, active metals catalysts such as palladium (Pd) [20,22,23,24,25,26,27], rhodium (Rh) [4,27,28,29], platinum (Pt) [29,30,31,32], and nickel (Ni) [20,26] have been applied either in bulk or in conjunction with support. In particular, elemental Pd has an excellent H_2_-adsorption capacity and a selectivity and stability that are superior to other metal catalysts, making it suitable for aqueous HDC reactions under moderate conditions [25,33,34]. 

Recent studies have focused on catalyst supports such as alumina [22,35,36,37], titania [38,39], and carbon-based materials [4,23,26,40,41,42] to improve both catalytic activity and stability. More recently, graphene-based materials such as graphene oxide (GO) and reduced graphene oxide (rGO) have been introduced in various environmental applications, for example, as an adsorbent [43] or absorbent [44], as well as employing as a support for Pd catalysts, with the presence of oxygen groups in the GO and rGO serving as anchoring and nucleation sites for the growth of metal particles and the immobilization of Pd catalysts [45,46,47]. However, it remains a significant challenge to develop a highly active, recyclable system using existing Pd catalysts for the HDC of CPs under mild conditions [48,49,50,51,52,53]. 

To address this limitation, we prepared, in the present study, Pd catalysts deposited on inorganic (i.e., alumina) and carbonaceous (i.e., GO and rGO) support materials in order to understand how the support influences the dispersion and electronic properties of the Pd. After observing the structural and morphological characteristics of the Pd catalysts, their HDC performance in terms of CP removal was compared and optimized at ambient temperature and with atmospheric pressure. The Pd-based catalysts were also granulated in order to improve their recyclability and promote in-situ catalytic HDC, which has not previously been reported in previous studies.

In the present study, 4-chlorophenol (4-CP) was selected as the target pollutant to evaluate the influence of operating factors on the aqueous HDC reaction using Pd-based catalysts. Alumina, GO and rGO were selected as support materials for the Pd-based catalysts due to their unique physicochemical characteristics. The effects of catalyst dosage, initial 4-CP concentration, and pH on 4-CP removal were subsequently evaluated. Furthermore, granular catalysts were developed using sand in the catalytic HDC of 4-CP under optimized experimental conditions, which offers new insights into the Pd-catalyzed HDC process for the removal of CPs in practical applications. 

## 2. Materials and Methods

### 2.1. Reagents

A complete list of chemical reagents used in this study is provided in Text S1 in the Supplementary Materials. 

### 2.2. Synthesis of the Pd-Based Catalysts

#### 2.2.1. Synthesis of the Powdered Pd/Al Catalyst

The Pd/alumina (Pd/Al) catalyst was synthesized via a simple wet impregnation method [54]. First, the alumina was dried at 100 °C for 24 h. A Pd solution was then injected into the dried alumina to a set Pd loading of 1, 3, or 5 wt.% with labels of Pd/Al (1%), Pd/Al (3%), and Pd/Al (5%), respectively. After injection, the mixture was stirred at 300 rpm at room temperature for 2 h and then dried in an oven at 100 °C for 24 h to completely remove the moisture. Finally, the dried samples were calcinated at 350 °C for 2 h to allow the metal catalysts to adhere to the support. 

The catalyst was activated using NaBH_4_ before the catalytic reaction. The catalyst was dispersed in 15 mL DI water and stirred quickly at 700 rpm for 15 min. During stirring, 10 mL of 0.01 M NaBH_4_ were slowly added dropwise to activate the Pd. The reduced catalyst was separated by centrifugation for 10 min at 4000 rpm, washed twice with DI water in an anaerobic chamber to remove the residual chemicals, and used immediately for the batch experiments.

#### 2.2.2. Synthesis of the Powdered Pd/GO and Pd/rGO Catalysts

The GO and rGO used in the experiment were manufactured using a modified version of Hummer’s method [55,56], which is described in detail in Text S2. To prepare the Pd/GO and Pd/rGO catalysts, GO and rGO were added to DI water, and the slurry was sonicated for 2 h using a bath sonicator. Next, a Pd solution was added into either GO or rGO suspension and stirred for 6 h at 80 °C. With the addition of 0.1 M NaBH_4,_ Pd/GO and Pd/rGO were obtained by reducing Pd metal. The obtained catalysts were then calcinated in a furnace using a stream of nitrogen gas with a flow rate of 0.7 L/min at a temperature of 150 °C for 24 h.

#### 2.2.3. Granulation of Pd/GO and Pd/rGO with Sand to Prepare the Pd/GOSC and Pd/rGOSC Composites 

To prepare granular Pd/GO and Pd/rGO with sand as a support, the weight ratio of each of the catalysts and sand was set at 1:100. First, 0.05 g of the powdered catalyst was added to 500 mL DI water and sonicated for 2 h to disperse it evenly. The obtained slurry was mixed with 5 g of 50–70 mesh sand, stirred at 80 °C for 6 h, and dried in an oven for 24 h at 60 °C. The granulation process was conducted in a furnace using a stream of nitrogen gas with a flow rate of 0.7 L/min at a temperature of 150 °C for 24 h. The obtained granular Pd/GO and Pd/rGO with sand were labeled Pd/GOSC and Pd/rGOSC, respectively. Unlike Pd/rGOSC, Pd/GOSC was observed to form agglomerates and tended to separate from the sand. This was because both GO particles and sand were negatively charged over a wide pH range, creating electrical repulsion between them [57]. Therefore, only the catalytic performance of Pd/rGOSC was examined in subsequent experiments for the removal of 4-CP from water. 

### 2.3. Characterization Methods

The morphology, elemental composition, and elemental distribution of Pd/Al (Pd [5 wt.%]), Pd/GO (Pd [5 wt.%]), and Pd/rGO (Pd [5 wt.%]) were characterized using scanning electron microscopy (SEM; EVO10, Carl Zeiss, Oberkochen, Germany) coupled with energy dispersive spectrometry (EDS), with an accelerating voltage of 10 (or 15) kV. X-ray diffraction (XRD; Bruker DE/D8 Advance, Bruker, Billerica, MA, USA) was used to evaluate the crystalline structure of the synthesized Pd/Al catalyst. The scans were recorded using Cu Kα radiation at a voltage of 2.2 kV in the range of 10 to 80° (2θ) with a scan speed of 0.1 s/step. The structural differences between the Pd/GO and Pd/rGO in terms of their functional groups were also examined using Fourier transform infrared spectroscopy (FT-IR; Nicolet 6700, Thermo Fisher Scientific, Boston, MA, USA) with a KBr pellet in the range of 400 to 4000 cm^−1^, with a total of eight scans and a resolution of 4 cm^−1^. The Brunauer–Emmet–Teller (BET) surface area of the alumina and Pd/Al catalyst was further measured using 3FLEX (Micrometrics, Norcross, GA, USA), and the pore volume and diameters of the particles were determined using the Barrett–Joyner–Halenda (BJH) method.

To determine the stability of the catalysts before and after the HDC reaction, X-ray photoelectron spectroscopy (XPS; K-Alpha, Thermo Fisher Scientific, Boston, MA, USA) with a monochromatic Al X-ray source was used, and the accuracy of the binding energy measurements was ± 0.1 eV for all samples. An elemental analyzer (EA, Elementar, Macro-Cube/Micro-Cube, Langenselbold, Germany) was employed to confirm the elemental ratio of the granulated Pd/rGOSC composites. The amount of Pd loaded onto the surface of the catalysts before and after the HDC reaction was also estimated using inductively coupled plasma emission spectrometry (ICP-OES, Agilent 5110 SVDV, Agilent Technologies, Santa Clara, CA, USA). Prior to the analysis, fresh and used catalysts dissolved in aqua regia (HNO_3_ + HCl) were prepared using a low plasma induction system (microwave high-pressure distortion system, Anton-Paar, Multiwave 7000, Graz, Austria). 

Thermogravimetric studies the fresh and used Pd/rGO catalysts were conducted in a Shimadzu (DTG-60H, Shimadzu, Kyoto, Japan), with a heating rate of 10 °C/min in high purity flowing air atmosphere from room temperature to 800 °C. Approximately 4–11 mg of finely dried ground samples were heated in an open ceramic crucible. 

### 2.4. Catalytic Hydrodechlorination (HDC) Reaction for the Removal of 4-CP 

#### 2.4.1. HDC Reaction for the Removal of 4-CP Using Powdered Pd-Based Catalysts

The HDC reaction was carried out in a 1-L volume batch reactor that included a stirrer, sample injection/collection, a gas inlet, and a gas outlet [54]. Once the reactor was capped and well sealed, 50 cc/min of hydrogen gas was supplied to the reactor through a gas diffuser to produce oxygen-free conditions. Next, 0.5 g of the prepared Pd-based catalysts in powder form were added to the reactor through the sample injection unit and dispersed in DI water for 20 min before the reaction. Pd/GO and Pd/rGO were initially sonicated for 2 h before use. An appropriate volume of the 4-CP stock solution prepared at a concentration of 1000 mg/L was then directly added to the reactor to produce 4-CP concentrations of 25, 50, and 100 mg/L in the reactor. The effect of the initial solution pH was controlled using a carbonate buffer system with sodium carbonate and sodium bicarbonate powder (0.1 M of the final buffer concentration after addition) and 98% sulfuric acid. The solution with the catalyst was continuously stirred, and samples were periodically taken from the reactor (at 1, 3, 5, 10, 20, 30, 40, and 60 min), filtered using a 0.45-µm PES membrane filter, and analyzed using high-performance liquid chromatography (HPLC, Ultimate-3000, Thermo Fisher Scientific, Bonston, MA, USA). The samples taken from the mixture with Pd/GO or Pd/rGO were also centrifuged at 13,000 rpm for 3 min before filtration. Details of the conditions for HPLC are presented in Text S3. 

The 4-CP removal rate using the catalytic HDC process was calculated using Equation (1):

(1) $$4-{Chlorophenol}_{removal\;rate}=\frac{{\left[4-Chlorophenol\right]}_{initial}-{\left[4-Chlorophenol\right]}_{t}}{{\left[4-Chlorophenol\right]}_{initial}}$$

The HDC reaction for 4-CP removal was also interpreted according to pseudo-first-order reaction kinetics:

(2) $$lnC-lnC_{0}=-kt$$

#### 2.4.2. Continuous HDC Process Using a Granulated Pd/rGOSC Composite for 4-CP Removal

The catalytic performance of granulated Pd/rGOSC was initially evaluated prior to the continuous HDC process for the removal of 4-CP. Approximately 0.5 g of Pd/rGOSC was added to 50 mL of 4-CP solution (100 mg/L) and mixed at 30 rpm with the addition of 0.01 M NaBH_4_ solution. In this experiment, NaBH_4_ was used as a source of hydrogen (i.e., a reducing agent), as presented in Equation (3)[58]:

(3) $$NaBH_{4}\left(aq\right)+2H_{2}O(l)=4H_{2}(g)+NaBO_{2}(aq)$$

A continuous HDC experiment was conducted using a column filled with granular Pd/rGOSC. A schematic diagram of the HDC system setup is presented in Figure S1. Pd/rGOSC (10 g) was packed in the middle of a column (diameter 1.5 cm, length 20 cm, Pyrex), and the rest of the column was filled with 10–20 mesh sea sand. An aqueous 4-CP solution containing 0.05 M *NaBH*_4_ solution was fed continuously into the column at a flow rate of 0.2, 0.5, or 1 mL/min. Treated effluent samples were periodically taken from the reactor every 30 min, and the concentrations of 4-CP and phenol were determined using HPLC. At the same time, the column filled with 50–70 mesh sand instead of the catalyst was used as the control group in the experiment. 

## 3. Results and Discussion 

### 3.1. Structural Properties of the Powdered Pd-Based Catalysts (Pd/Al, Pd/GO, and Pd/rGO)

**Figure 1:**
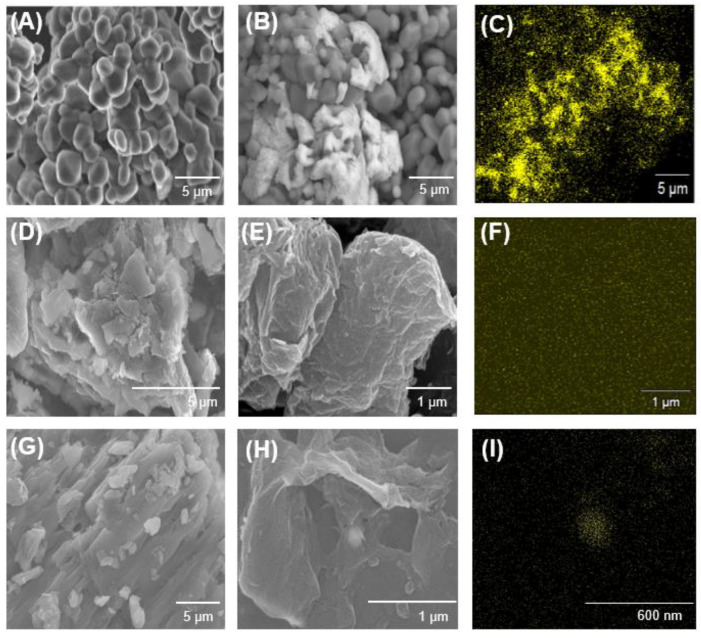
SEM images of powdered Pd/Al, Pd/GO, and Pd/rGO. (**A**) Alumina, (**B**) Pd/Al catalyst, (**C**) EDS-mapping image of Pd in Pd/Al, (**D**) GO, (**E**) Pd/GO catalyst, (**F**) EDS-mapping image of Pd in Pd/GO, (**G**) rGO, (**H**) Pd/rGO catalyst, and (**I**) EDS-mapping image of Pd in Pd/rGO.

SEM analysis was conducted to observe the surface morphology of the support materials (i.e., alumina, GO, and rGO) and the Pd-based catalysts (i.e., Pd/Al, Pd/GO, Pd/rGO) (Figure 1). The SEM images in Figure 1A,D,G show the presence of non-spherical particles of alumina, GO sheets, and stacked layers of rGO, respectively. The distribution of Pd on the surface of the catalysts is illustrated in Figure 1B,E,H. With the use of EDS analysis, aggregated Pd particles were observed in Pd/Al (Figure 1C), whereas Figure 1F,I shows a more even distribution of Pd immobilized on the GO and rGO because of the presence of functional groups on the surface of the support materials, which has also been reported in previous research [59]. 

The Pd/Al and Pd/rGO catalysts were taken in particular to examine their surface composition and the electronic state of each element due to their durability as well as superior electron collection and transportation compared to GO [60,61]. 

**Figure 2:**
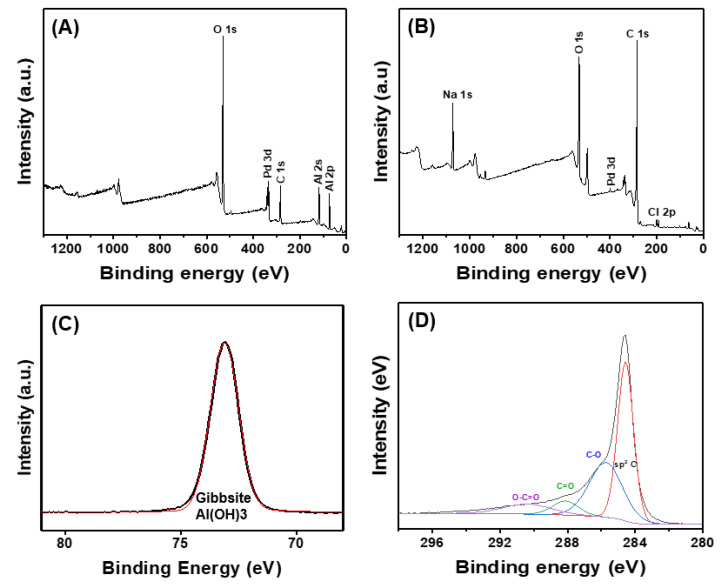
XPS analysis of powdered Pd/Al and Pd/rGO. XPS survey scan spectra of (**A**) Pd/Al and (**B**) Pd/rGO, (**C**) Al 2s XPS spectra acquired from Pd/Al, and (**D**) C 1s XPS spectra from Pd/rGO.

The XPS spectra for Pd/Al and Pd/rGO catalysts are presented in Figure 2. The presence of a Pd 3d peak in the Pd/Al spectra is clearly indicated in Figure 2A. As revealed by XRD analysis (Figure S2), the peaks at 2*θ* = 16.5° and 28.6°, which correspond to PdO (ICSD ID: 11992), indicated that Pd (in the form of PdO) was successfully loaded on the surface of the alumina (presented in the form of gibbsite, Al(OH)_3_, shown in Figure 2C). The nitrogen adsorption-desorption isotherm and the corresponding pore size distribution of the prepared Pd/Al catalyst were analyzed, as presented in Figure S3. The result showed hysteresis loops that belong to Type IV, as classified by IUPAC, which is associated with capillary condensation within mesopores [62]. The BET surface area and pore volume of the prepared Pd/Al catalyst were determined to be 0.8506 m^2^/g and 0.004826 cm^3^/g, respectively. Compared to the pristine alumina, the specific surface area of the Pd/Al was slightly increased, whereas the pore volume was decreased, which can be due to the loading of Pd. 

Similarly, the XPS survey scans for Pd/rGO indicate the presence of Pd, oxygen, and carbon on the rGO surface (Figure 2B). The C1s spectrum could be deconvoluted into four peaks associated with sp^2^ C-C, C-O, C=O, and HO-C=O in the range of 280–300 binding energy (eV). The surface oxygen-containing functional groups on the GO- and rGO-supported Pd catalysts were also analyzed using FT-IR (Figure S4). The lower intensity of the peaks associated with the oxygen-containing groups (e.g., the O-H stretching vibration at 3426 cm^−1^, C=O stretching and C-O stretching bands at 1706 cm^−1^ and 1069 cm^−1^, respectively, and the C=O vibrations and C-O group at the edges of GO at 1287 and 1040 cm^−1^, respectively) were observed for rGO. Low peak intensities of the oxygen-containing groups on the rGO surface are indicative of the successful reduction of GO [63]. Accordingly, based on this characterization, it was concluded that the desired Pd/Al and Pd/rGO catalysts were successfully obtained. 

### 3.2. Characterization of Granulated Pd/rGOSC 

**Figure 3:**
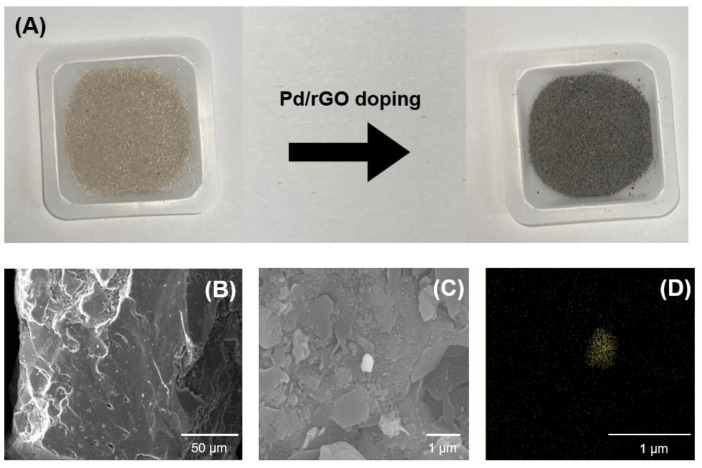
(**A**) Synthesis of Pd/rGOSC, SEM images of (**B**) sand and (**C**) Pd/rGOSC, and (**D**) SEM-EDS image of Pd/rGOSC.

To date, the practical application of powdered catalysts has been limited due to their low reusability and the requirement for post-separation processes (e.g., filtration) before the treated water can be released. To overcome these issues, Pd/rGO was employed in the present study to produce granulated Pd/rGOSC using sand. Figure 3A shows that, when Pd/rGO was added to the sand, the sand turned black. The surface of the sand was observed to be relatively smooth (Figure 3B), the result of rGO sheets from the Pd/rGO catalyst covering the surface (Figure 3C). The presence of Pd on the catalyst surface was also confirmed by EDS images (Figure 3D), indicating successful granulation. 

**Figure 4:**
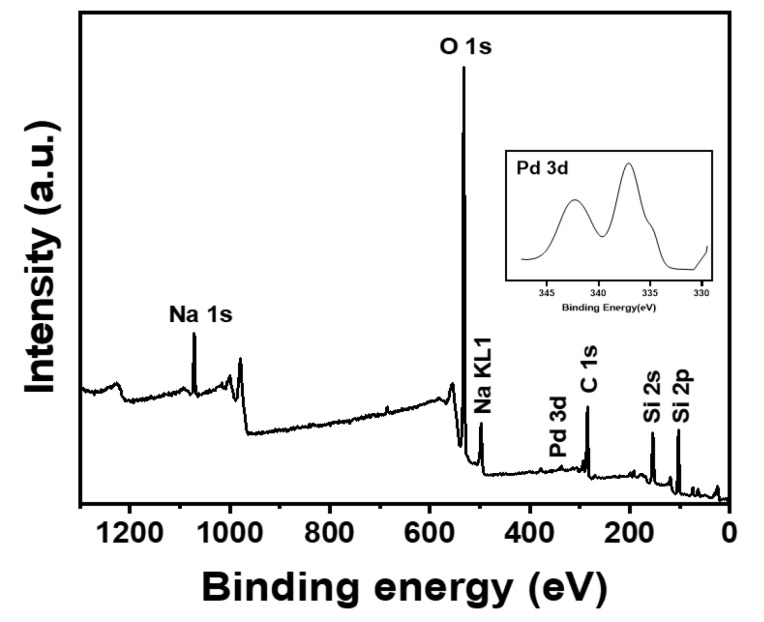
XPS survey scan spectrum for Pd/rGOSC.

Figure 4 presents XPS survey scans of the granulated Pd/rGOSC. The chemical composition of the Pd/rGOSC was similar to that of Pd/rGO except for the presence of an additional silica peak. A C 1s peak was observed due to the presence of Pd/rGO. A lower Pd 3d peak was observed in Pd/rGOSC because a ratio of Pd/rGO to the sand of 1:100 was used. Elemental analysis and ICP-OES analysis were employed to estimate the concentration of Pd in the Pd/rGOSC, which was calculated to be 0.0223% (Table S1). 

### 3.3. HDC of 4-Chlorophenol (4-CP) Using Pd/Al and Pd/rGO Catalysts

**Figure 5:**
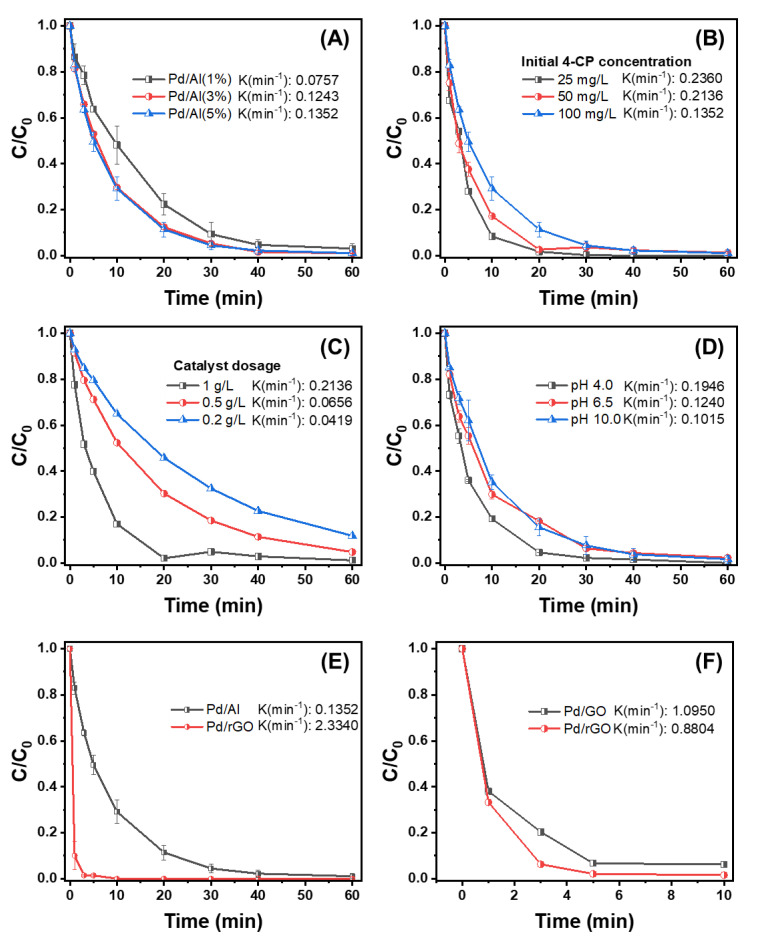
Degradation rates for 4-chlorophenol (4-CP) under different operating conditions. (**A**) Effect of Pd loading on Pd/Al catalyst ([4-CP]_0_ = 100 mg/L, catalyst dosage = 1 g/L), (**B**) initial concentration of 4−CP (catalyst dosage = 1 g/L, Pd/Al [5 wt.%]), (**C**) catalyst dosage (Pd/Al [5 wt.%], [4-CP]_0_ = 50 mg/L), (**D**) pH control (1 g/L of Pd/Al [5 wt.%], [4-CP]_0_ = 50 mg/L), (**E**) effect of the support material (Pd [5 wt.%] for Pd/Al and Pd/rGO, catalyst dosage = 1 g/L, [4-CP]_0_ = 100 mg/L), and (**F**) catalyst dosage control for Pd/GO and Pd/rGO (Pd [5 wt.%], catalyst dosage = 0.1 g/L, [4-CP]_0_ = 100 mg/L).

Based on the characterization of the catalysts, the removal of 4-CP via HDC using the desired Pd/Al and Pd/rGO catalysts was evaluated under different operating conditions in a batch reactor. Figure 5 shows the removal rate for 4-CP, which followed a first-order kinetic model, and a summary of the obtained kinetic reaction rates is presented in Table S2. 

#### 3.3.1. Effect of the Pd Load on the HDC of 4-CP

It was confirmed that alumina without Pd did not produce any catalytic reactivity for the removal of 4-CP (Figure S5). However, with the use of Pd/Al, 4-CP was effectively removed within a reaction time of 1 h. An increase in the Pd loading from 1% to 3% enhanced the removal rate, though no further enhancement was observed for 5% Pd (Figure 5A and Table S2). Based on these results, the higher the ratio of the Pd to the catalyst, the faster the reaction rate, because a higher Pd loading provides more reaction sites for the HDC reaction [64]. Because the reaction rate constants (*k*) for Pd/Al (3%) and Pd/Al (5%) (0.1243 min^−1^ and 0.1352 min^−1^, respectively) were not significantly different, Pd/Al (5%) was employed for subsequent experiments in relation to catalytic efficiency over cost effectiveness.

#### 3.3.2. Effect of the Initial 4-CP Concentration on the HDC of 4-CP

As presented in Figure 5B and Table S2, the effect of the initial 4-CP concentration on the 4-CP removal rate was investigated. Higher HDC reaction rates were achieved at a lower initial 4-CP concentration, with the highest reaction rate constant (0.2360 min^−1^) obtained for a 4-CP concentration of 25 mg/L. When the initial concentration was increased to 50 and 100 mg/L, the observed reaction rate constants decreased to 0.2136 and 0.1352 min^−1^, indicating that higher concentrations require longer reaction times [65]. 

#### 3.3.3. Effect of the Catalyst Dosage on the HDC of 4-CP

The results for Pd/Al (5%) dosages between 0.2 and 1 g/L are presented in Figure 5C and summarized in Table S2. Within this range, higher catalyst dosages led to a higher 4-CP removal rate. Most of the target compound was removed within 40 min with a catalyst dosage of 1 g/L, while only 88.2% and 95.2% were removed with 0.2 and 0.5 g/L, respectively. For the extended reaction time (60 min), more than 95% of the 4-CP was removed by catalyst dosages of 0.2 and 0.5 g/L. A higher catalyst dosage provides more reaction sites for HDC, thus enhancing the reaction rate [66]. 

#### 3.3.4. Effect of pH on the HDC of 4-CP

Figure 5D and Table S2 present the effect of the initial pH on the degradation rate of 4-CP using 1 g/L Pd/Al (5%). The reaction rate was generally higher with a lower pH. The removal efficiency for 4-CP was lower at pH 10 (81.74% removed within 20 min), while almost total removal was achieved at pH 4.5. This is a result of the large number of hydrogen atoms generated from alumina in an acidic medium and the reduction of Pd (Pd^n+^) in the catalyst under H_2_ generation, leading to the more rapid replacement of chloride by hydrogen. At a pH of 5 or higher, the surface of alumina is not completely oxidized [67,68] and, as the pH increases, a higher concentration of hydroxides can form, passivizing the surface layers of the catalyst and blocking the active sites of the Pd/Al, limiting HDC reactivity [5,69]. 

#### 3.3.5. Effect of the Catalyst Support Materials 

The effect of the catalyst supports on the HDC of 4-CP was evaluated using Pd/Al and Pd/rGO (Figure 5E and Table S2). The rate constant *k* for Pd in Pd/rGO was several times higher than that in Pd/Al, which can be ascribed to the high electrical conductivity, high chemical and thermal stability, and large surface area of rGO [45]. Ren et al. (2014) argued that the functional groups on the surface of graphene prevent the aggregation of metal particles, increasing the efficiency of the HDC reaction [41]. In addition, rGO can enhance the HDC reaction because it has a high affinity for phenolic compounds through the π bond between the phenol rings [70,71]. Since the fast reaction rates with the use of Pd doped on the graphene-based supports (e.g., Pd/GO and Pd/rGO catalysts) were observed, further experiments were conducted with a 1/10 lower dosage of GO and rGO, obtaining the higher reaction rates of *k* = 0.8804 min^−1^ and *k* = 1.0950 min^−1^, respectively. The faster reaction rate of Pd/rGO over Pd/Al is dependent on the property of rGO, such as faster electron acceptance and transport [60]. The retaining oxygen functional groups of rGO can avoid the agglomeration and leaching of Pd nanoparticles through interaction between the Pd nanoparticles and the graphene sheets; thus, higher catalytic of the Pd/rGO could be expected [59]. The catalytic capacity of the synthesized Pd/Al and Pd/rGO catalysts were further taken to compare with other catalysts reported in the previous studies, and similar or even higher reaction rates were obtained than previously reported catalysts (Table 1). 

Overall, the results of the present study revealed that the Pd loading, catalyst dosage, initial 4-CP concentration, initial pH, and support materials significantly affected the removal of 4-CP via the catalytic HDC process. In particular, the choice of support material had a significant effect on the catalytic activity of the Pd catalyst, which was also reported in the previous study, including Pd/C (1% *w*/*w*) and Pd/Al_2_O_3_ (1% *w*/*w*) [72]. The Pd/rGO catalyst produced the highest performance, with the 4-CP almost entirely removed during the first 5 min of the reaction, highlighting its excellent HDC ability.

### 3.4. Reusability of Pd/Al and Pd/rGO

**Figure 6:**
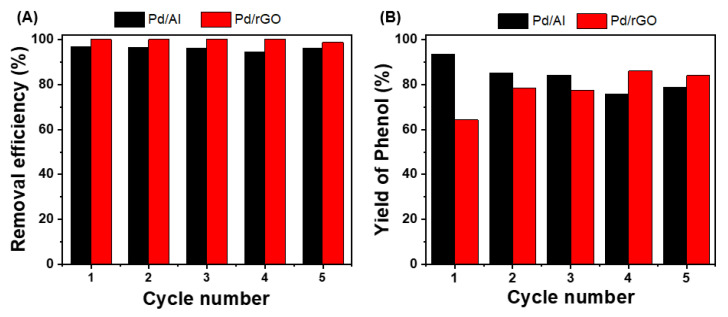
Reusability of (**A**) Pd/Al and Pd/rGO and (**B**) the phenol yield during the 4-CP catalytic HDC. The experimental conditions were Pd/Al and Pd/rGO dosage = 1 g/L and [4-CP]_0_ = 49.55 ± 3.98 for Pd/Al and 95.48 ± 8.24 mg/L for Pd/rGO.

By investigating the recycling and long-term use of the synthesized Pd-based catalysts, the active life and stability of the catalyst, which are essential criteria for the repetitive use of catalysts in environmental remediation processes using HDC, could be determined. First, recycling tests were conducted with powdered Pd/Al (5%) and Pd/rGO. After the HDC reaction, the catalysts were collected via filtration and washed with DI water. They were then re-used for HDC with the addition of a fresh 4-CP solution. This recycling process was conducted five times. Figure 6 shows that the removal rate for 4-CP was stable at 96.0 ± 0.008% and 99.7 ± 0.006% for Pd/Al and Pd/rGO, illustrating their high catalytic stability and no loss of activity in the 4-CP HDC process. There was a very slight decrease in the phenol yield, from 93.8% to 79.3%, after the five Pd/Al catalyst recycling runs. On the other hand, the phenol yield obtained from the Pd/rGO catalyst gradually increased, reaching 83.9% after five cycles, indicating its higher catalytic stability.

### 3.5. HDC in a Continuous Reactor Using Granulated Pd/rGOSC

**Figure 7:**
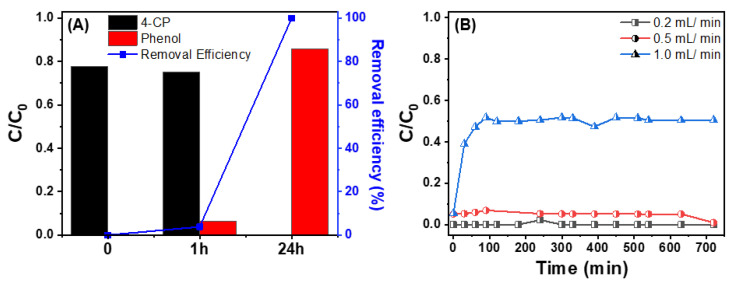
(**A**) Removal of 4-CP by granulated Pd/rGOSC in a batch reactor ([4-CP]_0_ = 50 mg/L, catalyst dosage = 10 g/L, Pd loading = 0.022%) and (**B**) concentration profiles of 4-CP in a continuous HDC process ([4-CP]_0_ = 63.9 ± 11.8 mg/L).

The catalytic HDC of 4-CP using granular Pd/rGOSC was evaluated in a batch reactor, and the results are presented in Figure 7A. It was confirmed that the 4-CP was reduced to phenol more slowly than with powdered Pd/rGO due to the lower catalyst loading of Pd/rGO in the granulated Pd/rGOSC (Pd/rGO:sand = 1:100). The yield of phenol increased to 10% within 1 h, and this rose to 100% after 24 h. 

Even though the reaction rate was lower, Pd/rGOSC was still able to effectively catalyze the HDC reaction for 4-CP removal. Therefore, we employed Pd/rGOSC in a continuous HDC process using a packed column in order to evaluate its activity and stability, and the column setting used for this study was shown in Figure S6. When the flow rate was set to 0.2 or 0.5 mL/min, the 4-CP removal rate was 90% or more after 12 h, while only 50% of the 4-CP was removed at a flow rate of 1 mL/min (Figure 7B and Figure S7). Thus, an optimal flow rate of 0.5 mL/min was employed for long-term operation testing using Pd/rGOSC. 

**Figure 8:**
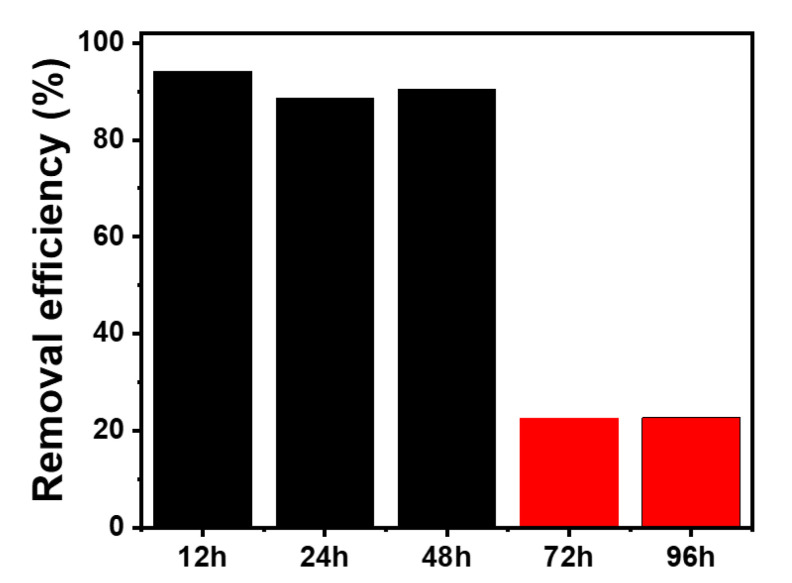
Removal efficiency for 4-CP during the HDC process with Pd/rGOSC.

The long-term stability of Pd/rGOSC was also evaluated for extended column operation of up to 96 h at a flow rate of 0.5 mL/min (Figure 8). It was found that more than 90% of the 4-CP was removed by the HDC reaction within 48 h, and the removal rate of 4-CP decreased sharply after 72 h. This indicates that the reactivation of the spent catalyst should be considered after two days of use. 

### 3.6. Proposed HDC Mechanisms Using Pd Catalysts for the Removal of 4-CP 

**Figure 9:**
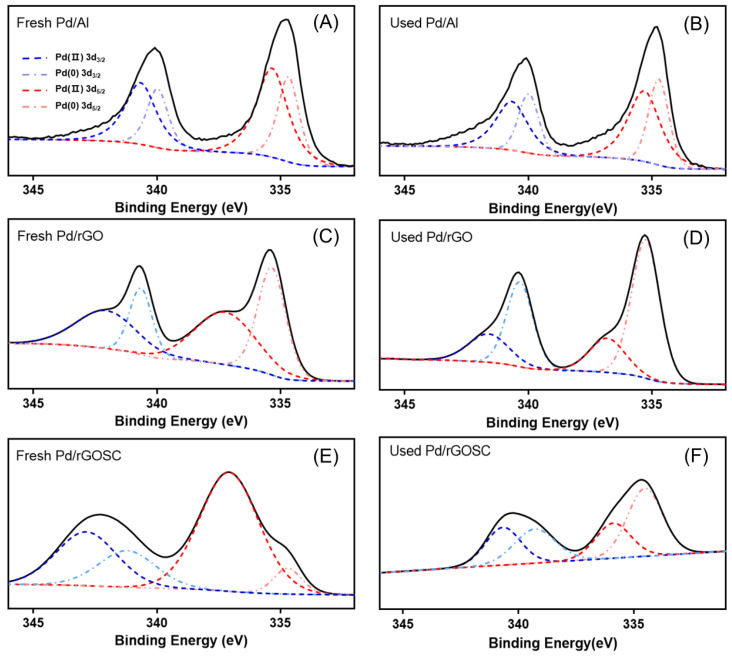
XPS analysis of powdered Pd/Al, Pd/rGO and Pd/rGOSC. Pd 3d XPS spectra acquired from (**A**) fresh Pd/Al, (**B**) used Pd/Al, (**C**) fresh Pd/rGO, (**D**) used Pd/rGO, (**E**) fresh Pd/rGOSC, and (**F**) used Pd/rGOSC.

In order to gain insight into the reaction mechanisms for 4-CP removal as well as the deactivation mechanism, mass balance data was analyzed, and a detailed XPS analysis of Pd in fresh and spent catalysts was conducted (Figure S8 and Figure 9, and Table 2). As a result, the mass balance of 4-CP and phenol showed more than 80%, indicating that phenol is the primary reaction product after substituting chloride for hydrogen.

Changes in the surface chemical composition of powdered Pd/Al (5%) and Pd/rGO were investigated, particularly their oxidation state after five cycles. Pd/rGOSC after 96 h of the HDC reaction was also compared to the fresh catalyst. 

In Figure 9A,B, sharp Pd^n+^ and metallic Pd^0^ peaks for both fresh and used Pd/Al were observed; after the HDC reaction, Pd^n+^ tended to be converted into Pd^0^, when the Pd metal catalyst reacted with H_2_. The binding energy of Pd 3d_3/2_ and Pd 3d_5/2_ in Pd/Al was 340.76 and 334.77 eV, which was somewhat similar to pure Pd (340.4 eV and 335.1 eV, respectively) [73], while the increase in the binding energy was clearly seen during the examination of Pd species in the fresh and used Pd/rGO catalyst (Figure 9C,D), with a sharp Pd^0^ peak observed. This may be due to the electron transfer from Pd to rGO, which was the result of the strong interaction between Pd and rGO. In contrast, Pd^n+^ exhibited a broad peak in Pd/rGO, indicating the presence of various electronic states due to the presence of various functional groups on the rGO surface. Figure 9E presents the Pd species in the fresh Pd/rGOSC, which were mainly found in the form of Pd^n+^ due to sufficient oxygen on the surface of rGO and SiO_2_, the main component of sand. For the catalyst after 96 h of use (Figure 9F), Pd^0^ was the most common form present. 

Table 2 also presents the ratio of Pd^n+^ to Pd^0^_,_ which affects the performance of the catalyst, with the reactivity highest when Pd^n+^/Pd^0^ is closer to 1 [33]. For the Pd/Al catalyst, it was observed that Pd^n+^/Pd^0^ decreased from 1.62 to 0.70 after the HDC reaction, while that for Pd/rGO fell significantly from 1.20 to 0.43 (Table 2). Ordóñez et al. (2003) reported that the interaction between a noble metal and its catalyst support is a crucial factor in determining the stability of a catalyst. As indicated by the Pd^n+^/Pd^0^ ratio, strong bonds between the Pd and alumina were formed, thus the Pd/Al was less affected than the Pd/rGO catalyst [74], even though both catalysts showed high stability during the HDC reaction, with almost unchanged 4-CP removal efficiency over five reaction cycles (see Figure 7). The strong interactions between Pd and Al can also be explained by the particle sizes of Pd nanoparticles of the Pd/Al, which was also seen in our BET result (Figure S3). Yuan et al. (2004) [72] showed the preferred leaching of larger Pd particles, affecting the loss of Pd content and resulting in the deactivation of catalysts. Given the consideration, the deactivation of Pd/rGO by the loss of Pd could be involved. Based on the measured Pd contents (%) on the surface of Al and rGO support before and after five cycles of the HDC reaction by XPS analysis (summarized in Figure S9 and Table S3), significant Pd nanoparticles loss was observed for Pd/rGO compared with Pd/Al. Additionally, thermogravimetric analysis of the fresh and used Pd/rGO catalysts showed two major mass losses at two different temperature regions (at 312–314 °C and 400–600 °C), presented in Figure S10. The weight loss peak at 312 and 314 °C is attributed to the characteristic peak for rGO, indicating the decomposition of oxygen containing functional groups (hydroxyl and carboxyl, etc.) for the fresh and used Pd/rGO. Another peak corresponds to the significant weight loss of graphene sheets observed for the fresh Pd/rGO at 527 °C, which goes down to 450 °C for the used Pd/rGO after five cycles of the HDC reaction. The decreased combustion temperature of graphene from the used Pd/rGO can be associated with the decomposition of the reactants deposits on the graphene sheet, the Pd/rGO catalyst bears the adsorption of 4-CP to some extent, and the presence of chlorine atoms in the carbonaceous deposits could cause the deactivation of the catalyst during the HDC reaction.

In spite of a large amount of Pd loss on the Pd/rGO as well as the presence of reactants in the graphene support, the catalytic performance of Pd/rGO continued over the five cycles. Tentatively, the Pd/rGO catalytic activity can be attributed to the strong metal-support interaction with an electron-deficient state, and the effect of particle size of the catalyst on the HDC reaction should be further examined.

Compared to the other catalysts, the Pd^n+^/Pd^0^ ratio for Pd/rGOSC exhibited the most significant changes. There are two potential reasons for the dramatic decrease in the removal rate after 72 h with the use of Pd/rGOSC. The first reason is associated with the electronic form of Pd. It was observed that the Pd in Pd/rGOSC mainly exists in the form of Pd^n+^ due to sufficient oxygen on the surface of rGO and SiO_2_. As the reaction proceeded, the Pd^n+^ was converted into Pd^0^, as observed in the XPS analysis (Table 2). Both Pd^n+^ and Pd^0^ participate in the HDC reaction, but it has been reported that a higher level of Pd^0^ compared to Pd^n+^ leads to a more rapid decrease in the performance of a catalyst [33]. The other possible reason is the loss of Pd particles during the continuous HDC process. Due to the XPS analysis detection limit to determine the Pd composition (%) in Pd/rGOSC, Pd/rGOSC was further analyzed by EA and ICP-OES before and after the reaction; a loss of 28.6% occurred for C and 60.2% for Pd (Table S1). Although a direct comparison cannot be performed between Pd/rGO and Pd/rGOSC, it was obvious that a significant loss of Pd nanoparticles occurred. This is again ascribed to the dramatic change in the Pd^n+^/Pd^0^ ratio. In the case of Pd/rGO and Pd/rGOSC, the loss of Pd particles on the surface, the support could be highly associated with the deactivation of the catalyst during the HDC reaction after 48 h. 

**Figure 10:**
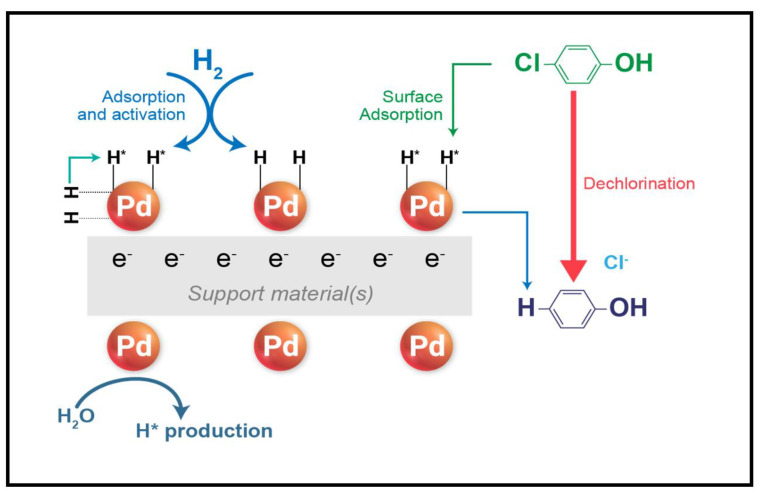
Proposed mechanisms for the 4-CP HDC reaction using a Pd catalyst.

Because the HDC reaction occurs on the surface of Pd catalysts, the HDC efficiency of these catalysts can be strongly affected by the pH, which has a critical effect on the hydrogen species and stability of the catalyst. In addition, the support materials affect the dispersion of the Pd catalyst in terms of the change in particle sizes and the reaction rate due to their physicochemical properties. Based on the obtained results, it can be concluded that the HDC reaction occurs through a chain reaction between the support materials, Pd catalyst, and hydrogen. An overview of the catalytic HDC reaction for the removal of 4-CP is presented in Figure 10 and Equations (4)–(8).

S + nH^+^ → S^n+^ + ne^−^ (4)

2H_2_O + 2e^−^ + Pd → 2H*_ads_ Pd + 2OH^−^ (5)

R−Cl + Pd ↔ (R−Cl) _ads_ Pd (6)

2H*_ads_ Pd + (R−Cl) _ads_ Pd → (R−H) _ads_ Pd + Cl^−^ + H^+^ (7)

(R−H) _ads_ Pd ↔ R−H + Pd (8)

In the first step, oxidation of the support material (S^0^) occurs to form S^n+^ (Equation (4)). The Pd catalyst then receives the electrons generated by the support materials and enters a complex reaction with the H_2_O (Equation (5)). Simultaneously, externally added H_2_ gas (from NaBH_4_ as a reducing agent) is adsorbed onto the surface of Pd from the solution and is reduced to form atomic hydrogen (H^*^) through the hydrogen-bonding network (Equation (6)). The target pollutant, 4-CP, is also adsorbed onto the surface of the Pd particles, the C-Cl bonds of 4-CP become unstable due to the resonance of the *p* electron orbital on Cl, and the cleavage of the R-Cl bond occurs via the replacement of the Cl atom with a hydrogen atom in the resonating phenol molecules (Equation (7)). As a result, the phenol molecules generated from 4-CP become dissociated from the surface of the Pd catalyst (Equation (8)) [19,20,75]. 

## 4. Conclusions

The present study investigated the catalytic HDC reaction for 4-CP using Pd catalysts prepared on various support materials. The Pd catalysts loaded on either alumina (Pd/Al) or graphene-based materials (Pd/GO and Pd/rGO) followed a first-order kinetic model, with variation in the operating conditions that affect the HDC reaction rates. Higher Pd loadings enhanced the HDC reaction due to a greater number of reaction sites, though an excessive amount of Pd did not improve the 4-CP HDC reaction. The pH of the solution significantly influenced the HDC of 4-CP, with an acidic pH (4.5 in the present study) providing more rapid dechlorination within the first 20 min due to the presence of hydrogen. In addition, the Pd catalysts supported by GO or rGO exhibited HDC reaction rates that were several times higher than those of Pd/Al, and complete dechlorination was obtained within 40 min. This enhanced HDC reaction can be explained by the physicochemical characteristics of graphene-related materials, mainly due to their ability to accept and transfer electrons. Granular Pd/rGO prepared using sand was also investigated in order to determine its ability to overcome current limitations of the HDC process using powdered catalysts in practical applications. An HDC efficiency of 90% for 4-CP was achieved after 48 h of continuous HDC, even in the presence of oxygen. Although the reusability and long-term stability of granular Pd/rGO fell after 72 h, which requires further improvement, the present study highlights the practicality and effectiveness of the use of Pd catalysts with graphene-based support materials in an HDC system. 

## Figures and Tables

**Table 1:** Comparison study between our study and previously reported catalysts.

Catalyst (%)	Support Material	4-CP Concentration	Catalyst Dosage	Temperature (°C)	*k* Value (min^−1^)	Ref
Pd (1%)	Pillared clay	0.1 g/L	1 g/L	25	0.0141	[27]
Pd (1%)	Al_2_O_3_	0.2 g/L	2 g/L	200	0.088	[36]
Pd (1.5%)	rGO	0.1 g/L	0.33 g/L	30	0.0564	[45]
Pd (0.5%) *	Al_2_O_3_	0.1 g/L	3.25 g/L	30	0.0167	[69]
Pd (1%)	Al	0.1 g/L	1 g/L	25	0.0757	This study
Pd (5%)	Al rGO	0.1 g/L 0.1 g/L	1 g/L 0.1 g/L	25	0.1352 2.3340	This study

* continuous flow.

**Table 2:** Summary of deconvoluted XPS spectra of Pd^0^ and Pd^n+^ and the Pd^n+^/Pd^0^ ratio from fresh and used catalysts.

	B.E of Pd (II) 3d_3/2_ (eV) [% Area]	B.E of Pd (0) 3d_3/2_ (eV) [% Area]	B.E of Pd (II) 3d_5/2_ (eV) [% Area]	B.E of Pd (0) 3d_5/2_ (eV) [% Area]	Pd^n+^/Pd^0^
Fresh Pd/Al	342.68	340.76	337.68	334.77	1.62
	[22.15]	[13.70]	[32.01]	[19.80]	
Used Pd/Al	342.19	339.98	337.04	334.71	0.70
	[7.85]	[19.18]	[12.20]	[27.80]	
Fresh Pd/rGO	341.48 [22.90]	340.58 [15.06]	337.18 [31.64]	335.42 [30.39]	1.20
Used Pd/rGO	340.88	340.28	337.07	335.23	0.43
	[13.06]	[28.48]	[16.77]	[41.69]	
Fresh Pd/rGOSC	342.49	340.08	337.28	334.78	4.05
	[24.73]	[14.64]	[55.48]	[5.20]	
Used Pd/rGOSC	342.38	338.88	336.38	334.18	0.69
	[21.26]	[21.44]	[19.70]	[37.61]	

## Data Availability

The data presented in this study are available on request from the corresponding author.

## Supplementary Materials

The following supporting information can be downloaded at: https://www.mdpi.com/article/10.3390/nano13091564/s1. Figure S1: A schematic diagram of a column test with continuous HDC process; Figure S2: XRD patterns of alumina and Pd/Al catalyst (Pd [5 wt.%]); Figure S3: N_2_ adsorption-desorption isotherms and BJH pore size distribution for alumina and Pd/Al catalyst (Pd [5 wt.%]); Figure S4: FT-IR spectra of reduced graphene oxide (rGO) and graphene oxide (GO); Figure S5: Concentration profile of 4-CP without palladium catalyst; Figure S6: Column test configuration (flow rate of 1 mL/min and 0.5 ml/min); Figure S7: Continuous reaction test result—phenol production; Figure S8: Mass balance of phenol and 4-chlorophenol using (A) Pd/Al catalyst and (B) Pd/rGO catalyst; Figure S9: XPS survey scan spectra for spent (A) Pd/Al (5%), (B) Pd/rGO (Pd [5 wt.%]), and (C) Pd/rGOSC after five cycles of the HDC reaction; Figure S10: TGA and DTA profiles of (A) fresh Pd/rGO (Pd [5 wt.%]) and (B) used Pd/rGO catalyst after five cycles of the HDC reaction; Table S1: Carbon and palladium composition in Pd/rGOSC using Elemental analysis and ICP-OES analysis; Table S2: Percentage of 4-CP removal, and pseudo first-order rate constant for HDC reaction of 4-chlorophenol for each parameter; Table S3: Raw and used Pd composition (%) in the catalyst using the XPS analysis; Text S1: List of chemical reagents; Text S2: Synthesis of GO and rGO by Hummer’s method; and Text S3: HPLC method for detection of 4-CP. The reference [55,56] have been cited in Supplementary Materials.
