# Supplementary material for: Catalytic Hydrodechlorination of 4-Chlorophenol by Palladium-Based Catalyst Supported on Alumina and Graphene Materials
Source: Nanomaterials (Basel). 2023 May 6;13(9):1564. doi: 10.3390/nano13091564 (PMC10181078; doi:10.3390/nano13091564)
Supplement: Supplementary file 1 [file nanomaterials-13-01564-s001.zip › nanomaterials-2366347-supplementary.pdf]

Supplementary Material

Catalytic hydrodechlorination of 4-chlorophenol by palladium-based catalyst supported on alumina and graphene materials

Jintae Jeon, Yuri Park*, Yuhoon Hwang*

Department of Environmental Engineering, Seoul National University of Science and Technology, Seoul 01811, Republic of Korea

*Corresponding author
Dr. Yuri Park
E-mail: yuripark@seoultech.ac.kr; Tel: +82-2-970-6626; Fax: +82-2-971-5776

Dr. Yuhoon Hwang
E-mail:yhhwang@seoultech.ac.kr; Tel: +82-2-970-6626; Fax: +82-2-971-5776

Text S1. List of chemical reagents

Palladium chloride ($PdCl_2$, Samchun Pure Chemical Co., Ltd., Korea) was used as a precursor for Pd based catalysts. As supporting materials for Pd catalyst, aluminum oxide (γ-Al_2O_3, Kanto Chemical Co., INC., Japan), GO, and rGO were used. For GO preparation, graphite powder (<20 μm; Sigma-Aldrich, USA), potassium permanganate (99.3%; Duksan Pure Chemicals Inc., Korea), sulfuric acid (98%; Daejung Chemicals & Metals Co., Ltd., Korea), phosphoric acid (85%, Samchun Pure Chemical Co., Ltd., Korea), hydrogen peroxide (34.5%; Samchun Pure Chemical Co., Ltd., Korea), and ethyl alcohol (94.5%; Daejung Chemicals & Metals Co., Ltd., Korea) were used. For the preparation of rGO, sodium borohydride (98%; Samchun Pure Chemical Co., Ltd., Korea) and ethylene glycol anhydrous (99.8%; Sigma Aldrich, USA) were used. For granulation of Pd/rGO, sand (50 – 70 mesh) and sea sand (10 – 20 mesh) were purchased from Sigma Aldrich (USA) and Daejung Chemicals & Metals Co., Ltd. (Korea), respectively and used as supplied.

4-chlorophenol (99%; Samchun Pure Chemical Co., Ltd., Korea), phenol (99%; Daejung Chemicals & Metals Co., Ltd., Korea) were used for HDC reaction. The pH control was done by 0.1M sodium bicarbonate (99.5%; Sigma Aldrich (USA)) and 0.1M sodium carbonate (99.7%; Sigma Aldrich (USA)). Methanol (HPLC grade; Samchun Pure Chemical Co., Ltd., Korea) and acetic acid (HPLC grade; Samchun Pure Chemical Co., Ltd., Korea) were used for HPLC analysis. DI water was generated using a Milli-Q system (Synergy®, Merck Millipore, USA).

Text S2. Synthesis of GO and rGO by Hummer's method

GO was produced using the modified Hummer's method. A mixture of 3 g of graphite and 18 g of Potassium permanganate was slowly added to 400 mL of a solution made of H_2SO_4 (360 mL)/H_3PO_4 (40 mL) and stirred at 50 °C for 12 h. Then, the solution prepared in 400 mL of pre-frozen DI water was slowly poured. After pouring the solution, 3 mL of H_2O_2 (34.5%) was injected, mixed lightly, and stored to overnight. At this time, it was confirmed that a yellow layer was formed, and GO was successfully produced. Thereafter, washing was performed three times in DI water, HCl (10%) and ethanol and using a centrifuge set to 4000 rpm and 20 min. After washing, it was dried in a vacuum oven set at 60 °C for 12 h to prepare GO [55].

The production of rGO was carried out in a simple method using sodium borohydride as a reducing agent. 0.5 g of the produced GO was put in 250 mL DI water, sonicated for 2 h, and dispersed. Thereafter, 0.95 g of $NaBH_4$ was added to the water at 80 °C and stirred at 150 rpm to overnight. At this time, the concentration of $NaBH_4$ was added 0.3 M to induce a reduction reaction. After stirring for 12 h, the mixture was stirred in a centrifuge set to 4000 rpm for 20 min, washed using DI water, and dried in a vacuum oven set to 60 °C for 12 h to prepare rGO [56].

Text S3. HPLC Method for detection of 4-CP

The concentration of 4-CP was measured by UHPLC. The system, with Thermo LPG-3400SD gradient pump was used for all 4-CP samples taken after the HDC reaction. A Thermo Column C18 Accucore AQ (150×4.5 mm, 2.6 μm) was used and maintained at 50 °C throughout the run. The mobile phase of 0.1% acetic acid in DI water and 0.1% acetic acid in methanol (1:1 v/v) at the flow rate of 0.5 mL/min with total 15 mins run time was used and the UV detector wavelength set at 270 nm was used for the detection of 4-CP and phenol peaks.

Table S1. Carbon and palladium composition in Pd/rGOSC using Elemental analysis and ICP-OES analysis

Elements	Fresh Pd/rGOSC	Used Pd/rGOSC
Carbon (%)	0.14	0.10
Pd (ppm)	222.76	88.66

Table S2. Percentage of 4-CP removal, and pseudo first-order rate constant for HDC reaction of 4-Chlorophenol for each parameter

Parameter	Catalyst dosage(g/L)	4-CP C_i (mg/L)	Pd-loading (w.t%)	Removal rate (%)	K (min^{-1})	R^2
Support material						
Alumina	1	100	5	98.85	0.1352	0.9941
GO	1	100	5	100	3.075	0.9974

rGO	1	100	5	100	2.334	0.9975
			Pd-loading			
Pd 1%	1	100	1	96.94	0.0757	0.9899
Pd 3%	1	100	3	98.99	0.1243	0.9962
Pd 5%	1	100	5	98.85	0.1352	0.9941
			Initial concentration of 4-CP			
25	1	25	5	98.85	0.2360	0.9876
50	1	50	5	98.51	0.2136	0.9926
100	1	100	5	98.85	0.1352	0.9941
			Catalyst dosage			
0.2	0.2	50	5	88.17	0.0419	0.9984
0.5	0.5	50	5	95.21	0.0656	0.9989
1	1	50	5	98.51	0.2136	0.9926
			pH control			
4	1	50	5	98.63	0.1946	0.9921
6.5	1	50	5	98.42	0.1240	0.9904
10	1	50	5	97.23	0.1015	0.9916

Table S3. Raw and used Pd composition (%) in the catalyst using the XPS analysis

Pd(%)	Pd/Al	Pd/rGO	Pd/rGOSC
Fresh catalyst	2.11%	0.86%	-

Used catalyst	1.69%	-	-

Note- The Pd composition in the used Pd/rGO, and fresh & spent Pd/rGOSC were detected less than the detection limit of XPS analyzer that we used.

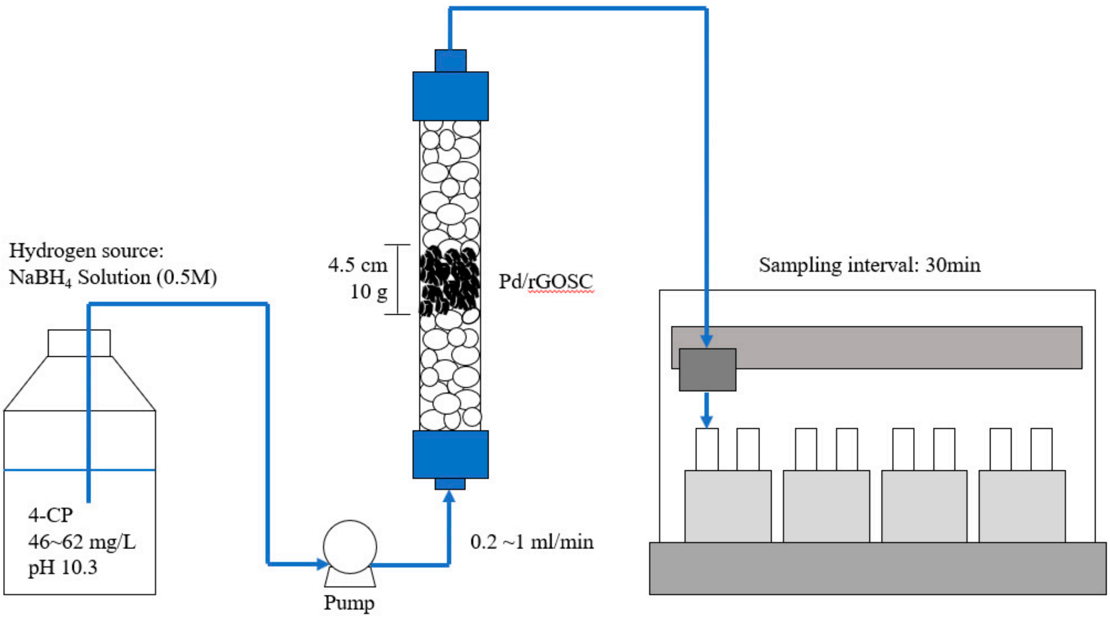

Figure S1. A schematic diagram of a column test with continuous HDC process

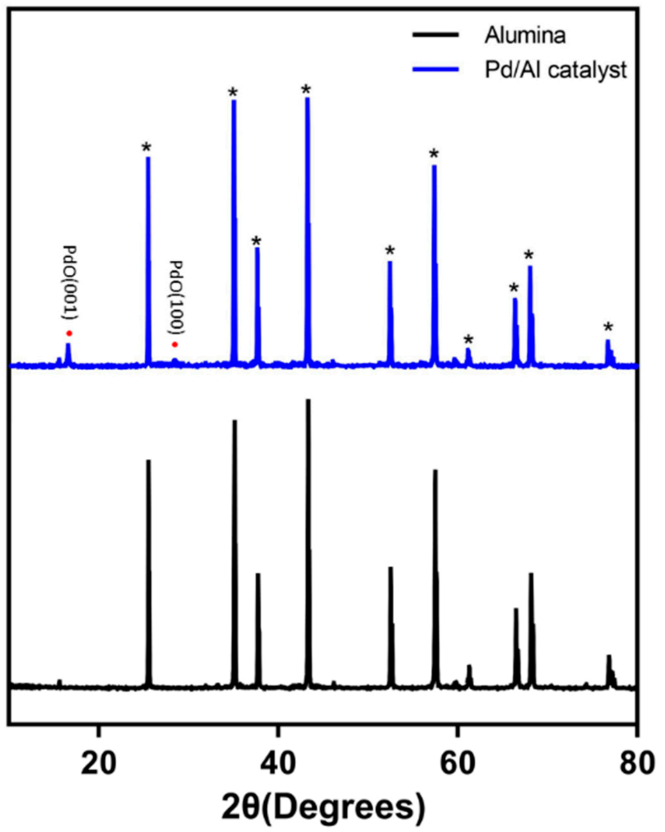

(*) Alumina x-ray diffraction peak

Figure S2. XRD patterns of alumina and Pd/Alumina catalyst (Pd [5 wt.%])

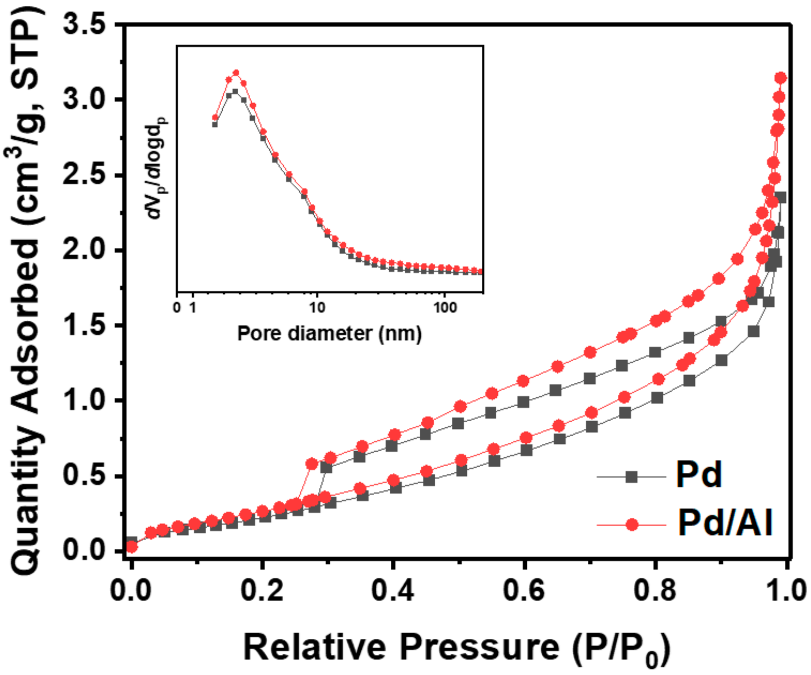

Figure S3. N_2 adsorption-desorption isotherms and BJH pore size distribution for alumina and Pd/Al catalyst (Pd [5 wt.%])

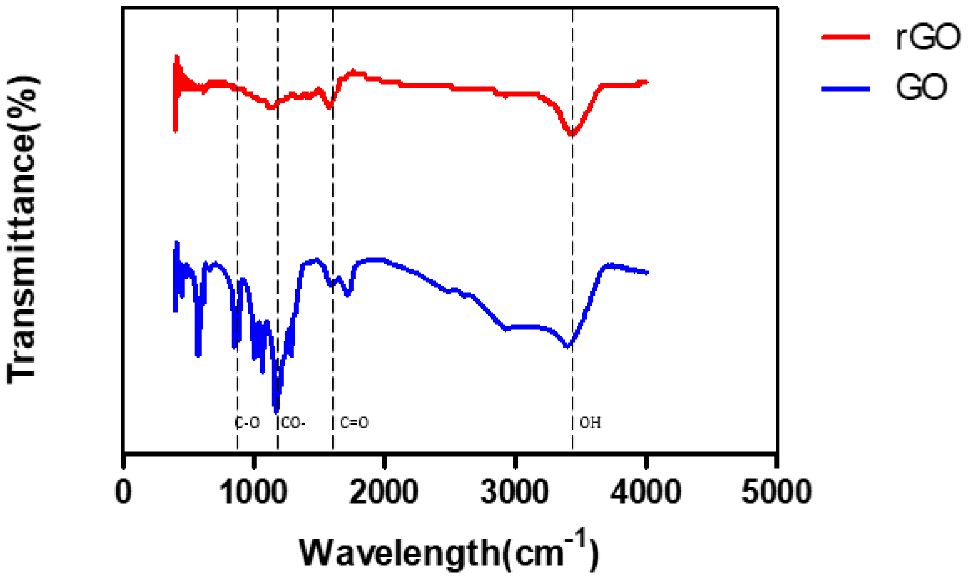

Figure S4. FT-IR spectra of reduced graphene oxide (rGO) and graphene oxide (GO)

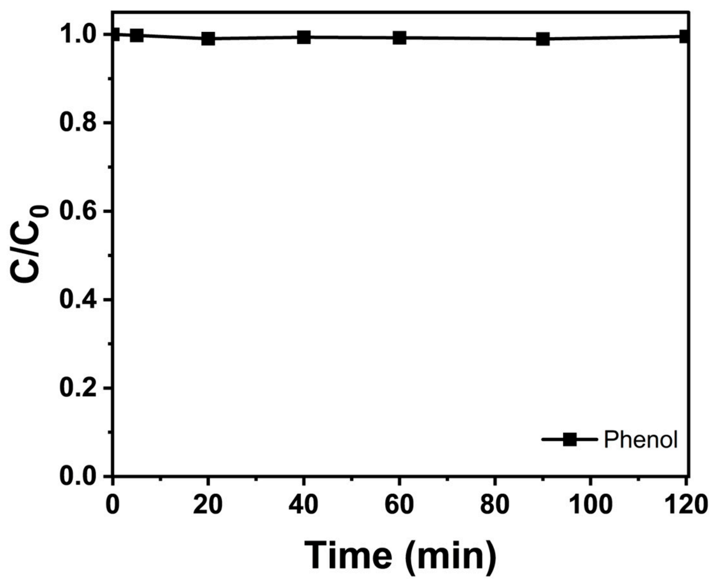

Figure S5. Concentration profile of 4-CP without Palladium catalyst

(Alumina dosage: 1 g/L, initial Concentration of 4-CP: 50 mg/L)

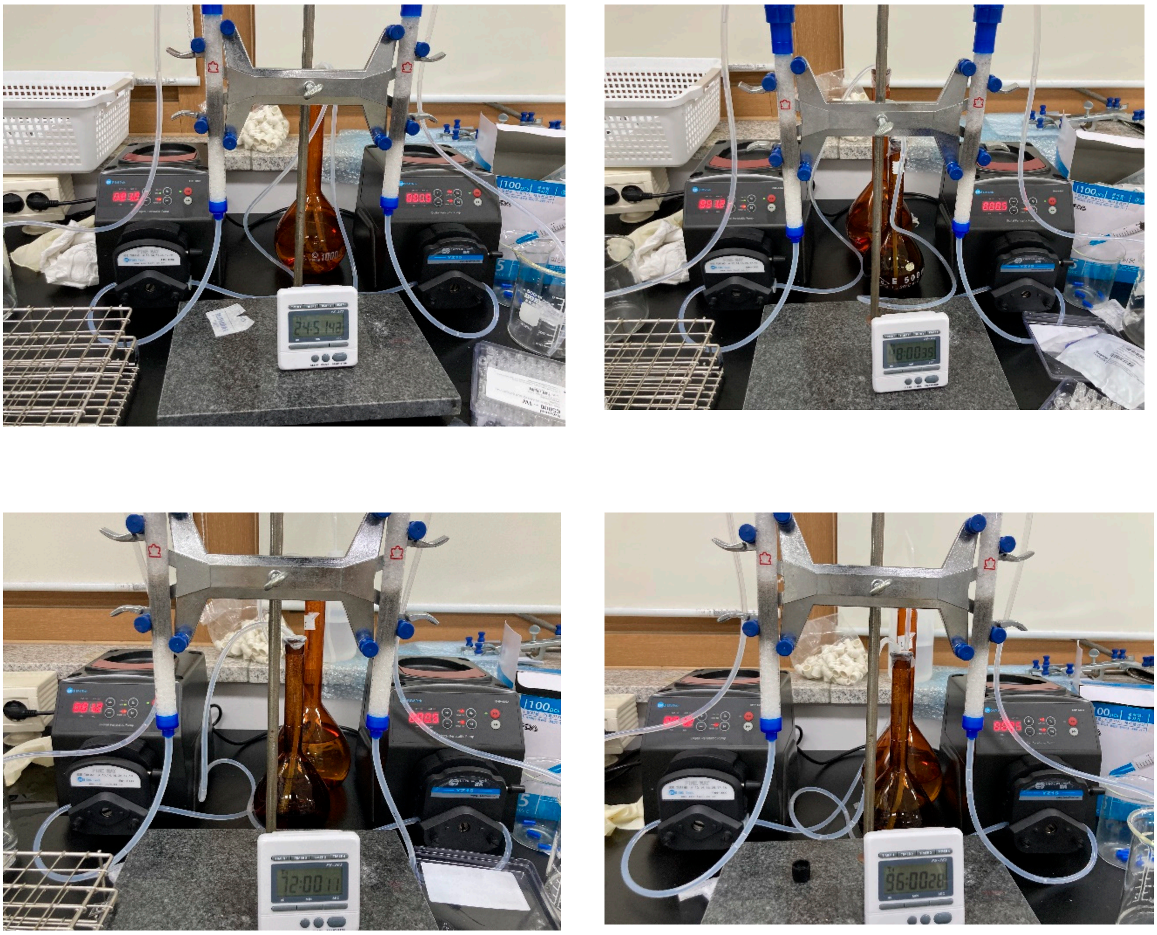

Figure S6. Column test configuration (flow rate: left: 1 mL/min, right: 0.5 mL/min)

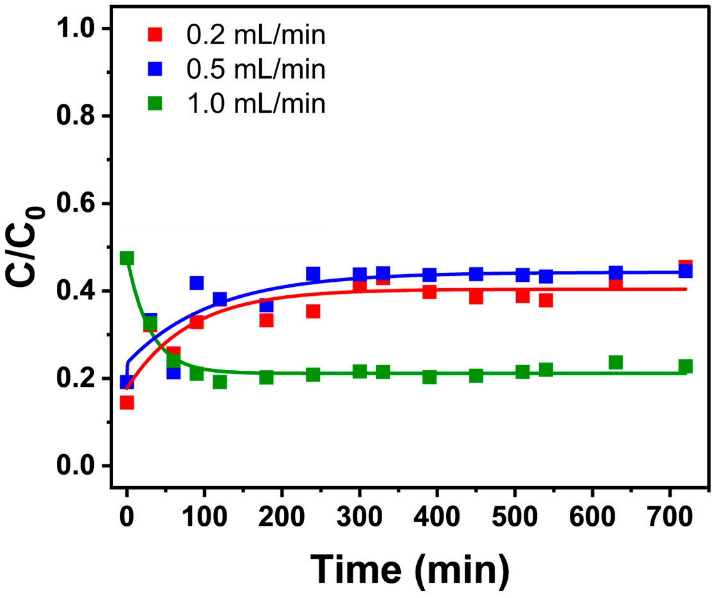

Figure S7. Continuous reaction test result – phenol production.

(4-CP initial concentration: 46~62 mg/L, catalyst dosage: 10g, □: Phenol)

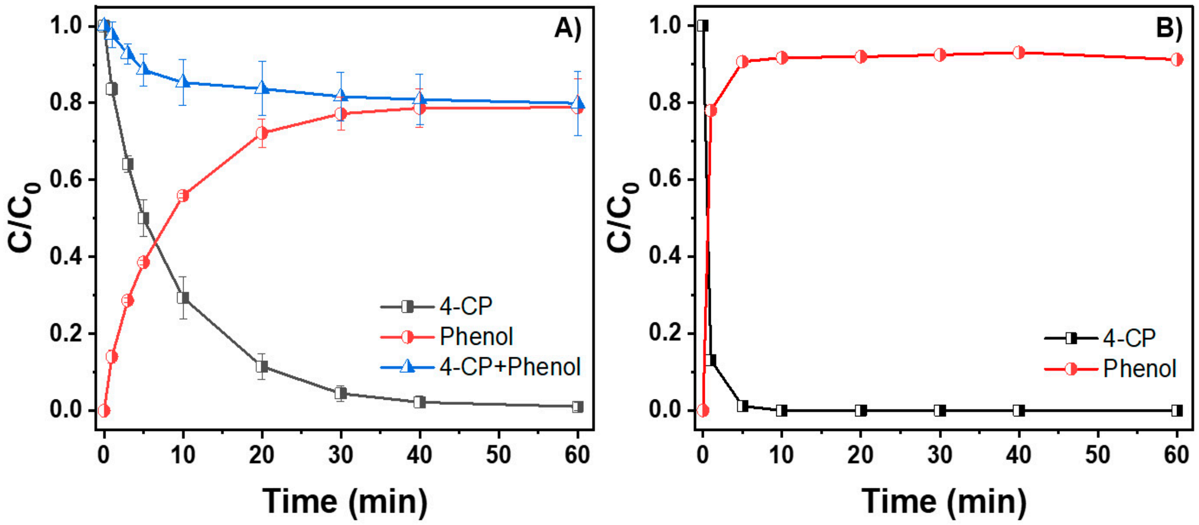

Figure S8. Mass balance of phenol and 4-chlorpohenol using (A) Pd/Al catalyst and (B) Pd/rGO catalyst

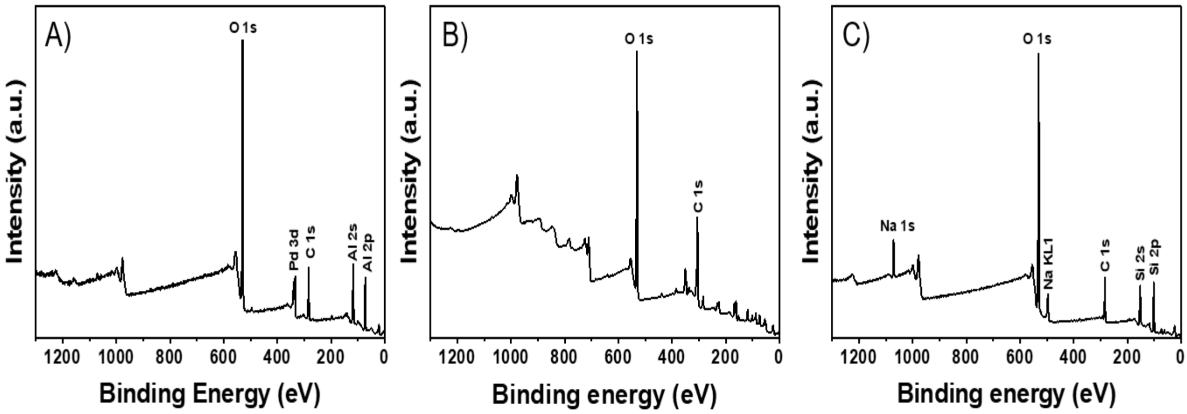

Figure S9. XPS survey scan spectra for spent (A) Pd/Al (5%), (B) Pd/rGO (Pd [5 wt.%]), and (C) Pd/rGOSC after five cycles of the HDC reaction

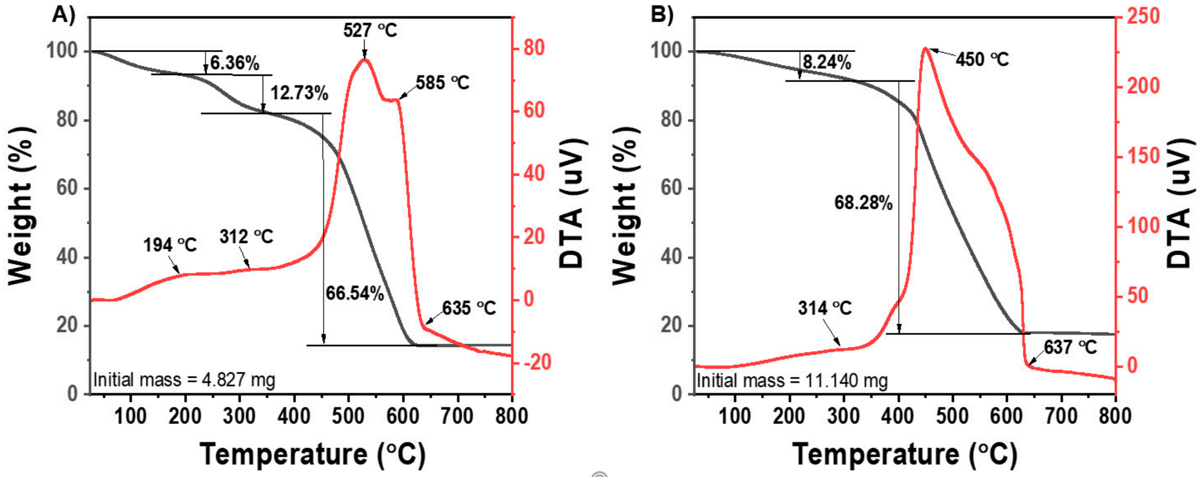

Figure S10. TGA and DTA profiles of (A) fresh Pd/rGO (Pd [5 wt.%]) and (B) used Pd/rGO (after five cycles of the HDC reaction) catalysts
